# Potential role of intermittent functioning of baroreflexes in the etiology of hypertension in spontaneously hypertensive rats

**DOI:** 10.1172/jci.insight.139789

**Published:** 2020-10-02

**Authors:** Feng Gu, E. Benjamin Randall, Steven Whitesall, Kimber Converso-Baran, Brian E. Carlson, Gregory D. Fink, Daniel E. Michele, Daniel A. Beard

**Affiliations:** 1 Department of Vascular Surgery, Second Xiangya Hospital, Central South University, Changsha, China.; 2 Department of Molecular and Integrative Physiology, University of Michigan, Ann Arbor, Michigan, USA.; 3 Department of Pharmacology and Toxicology, Michigan State University, East Lansing, Michigan, USA.

**Keywords:** Cardiology, Complex traits, Hypertension

## Abstract

The spontaneously hypertensive rat (SHR) is a genetic model of primary hypertension with an etiology that includes sympathetic overdrive. To elucidate the neurogenic mechanisms underlying the pathophysiology of this model, we analyzed the dynamic baroreflex response to spontaneous fluctuations in arterial pressure in conscious SHRs, as well as in the Wistar-Kyoto (WKY), the Dahl salt-sensitive, the Dahl salt-resistant, and the Sprague-Dawley rat. Observations revealed the existence of long intermittent periods (lasting up to several minutes) of engagement and disengagement of baroreflex control of heart rate. Analysis of these intermittent periods revealed a predictive relationship between increased mean arterial pressure and progressive baroreflex disengagement that was present in the SHR and WKY strains but absent in others. This relationship yielded the hypothesis that a lower proportion of engagement versus disengagement of the baroreflex in SHR compared with WKY contributes to the hypertension (or increased blood pressure) in SHR compared with WKY. Results of experiments using sinoaortic baroreceptor denervation were consistent with the hypothesis that dysfunction of the baroreflex contributes to the etiology of hypertension in the SHR. Thus, this study provides experimental evidence for the roles of the baroreflex in long-term arterial pressure regulation and in the etiology of primary hypertension in this animal model.

## Abstract 2

Baroreflex dysfunction contributes to the etiology of hypertension in a genetic model of primary hypertension.

## Graphical abstract

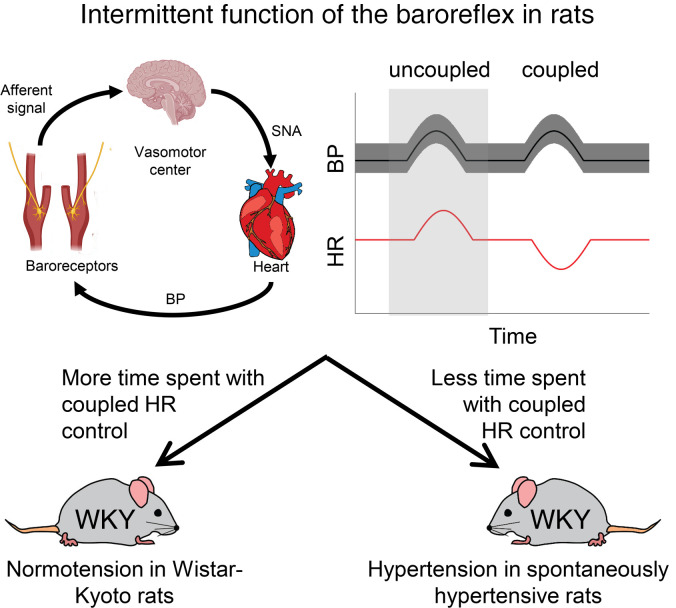

## Introduction

Physiological control of arterial blood pressure (BP) is achieved via the interaction of multiple organs and organ systems. The pressure waveforms in the systemic arteries are governed by the interactions between ventricular pumping and arterial mechanics, the ionotropic and chronotropic state of the heart, and the preloads driving filling of the left and right sides of the heart (1–4). These governing processes are in turn regulated by the autonomic nervous system and endocrine signals, notably the baroreflex and the renin-angiotensin-aldosterone system (5–8). Because each one of these systems — mechanical, autonomic, and endocrine — has a direct influence on the functions of the others, no single controller of arterial pressure or single root cause of primary hypertension has been identified (9). Rather, just as the physiological control of BP is a multifactorial systems-level phenomenon, it may be that the pathophysiology of hypertension is most generally understood as a multifactorial phenomenon. Moreover, because chronic increases in pressure can, in principle, both cause and be caused by mechanical remodeling and changes to autonomic and renal function (7, 10–13), it is possible that many different primary insults affecting different systems could all drive the system toward the same multifactorial pathological phenotype (14, 15).

One of the hypothesized specific etiologies for primary hypertension is the mechanogenic etiology (16, 17), where stiffening of large arteries reduces the sensitivity of baroreceptor afferents to the arterial pressure wave, requiring higher pressure changes to achieve a given level of input to the central controllers of autonomic state (18), chronically shifting the set point of the baroreflex to an elevated pressure (7, 11). To test this hypothesis, we assayed baroreflex function in the spontaneously hypertensive rat (SHR) and the Wistar-Kyoto (WKY) control strains during the development of the hypertensive phenotype in the SHR model over an 8-week period. Our goals were to relate changes in arterial mechanics, arterial pressure, and baroreflex function and determine the sequence of changes during the development of the phenotype.

Analyzing time-series data of arterial pressure and heart rate from these animals, we made a series of surprising observations. Rather than a simple proportional relationship between arterial vessel distensibility and baroreflex sensitivity, analysis of data from these animals revealed a markedly intermittent functioning of baroreflex-mediated control of heart rate (HR). The cardiovascular state of these animals appears to randomly and intermittently switch between long periods (up to several minutes) when the baroreflex-mediated control of HR is operating in an on state and long periods when it is operating in an off state. On states are identified as periods when BP and HR fluctuations are coupled in a way that changes in HR follow from preceding changes in mean arterial pressure (MAP). Off states are identified as periods when there is no coupling between fluctuations in BP and HR. Second, we observed that the fraction of time spent in the off state increased with age, was higher in SHR than in WKY rats, and was predictive of MAP.

Based on these observations, we hypothesized that the observed differences in intermittent functioning of the baroreflex play a causal role in determining differences in arterial pressure between the SHR and WKY rat. To test this hypothesis, we performed large-scale time-series analyses to determine: (a) relationships between on/off state and arterial pressure in SHR and WKY rats at 7, 10, and 15 weeks of age and (b) trends in MAP during on versus off state in these animals at the 3 different ages. Similar experiments were performed in Dahl salt-sensitive (SS), Dahl salt-resistant (SR), and Sprague-Dawley (SD) rats to determine the degree to which the observed phenomena are unique to the SHR/WKY model. Finally, baroreflex sinoaortic denervation studies were performed to further test the hypothesis that the greater proportion of engagement of the baroreflex is partially responsible for maintaining lower pressures in the WKY compared with the SHR.

Results reveal that associations between intermittent functioning of baroreflex control of HR and MAP are apparent in the SHR and WKY strains but not in other rat strains studied. Sinoaortic denervation resulted in an increase in MAP lasting several days in WKY rats but not in SHRs, consistent with the hypothesis that a difference in baroreflex function plays a role in determining the elevated MAP in the SHR compared with the WKY rat.

## Results

### Aortic pressure increases with age in SHR and WKY rats.

Arterial pressure and related phenotypes were observed in SHR and WKY rats during the development of the hypertensive phenotype in the SHR, a genetic model of primary hypertension (19). Conscious direct BP measurement during the whole dark cycle (6 p.m. to 6 a.m.) was taken in the SHR and WKY rats at 3 ages: (a) 7 weeks, when the MAP of the SHRs is expected to begin to rise; (b) 10 weeks, when the MAP in the SHRs is about halfway between the 7-week value and the maximum achieved around 14 weeks; and (c) 15 weeks, when the MAP progression is expected to have leveled off at an essentially constant MAP (20). We also employed a vessel ultrasound technique to measure the elasticity of the aorta and carotid arteries at the same times.

**Figure 1:**
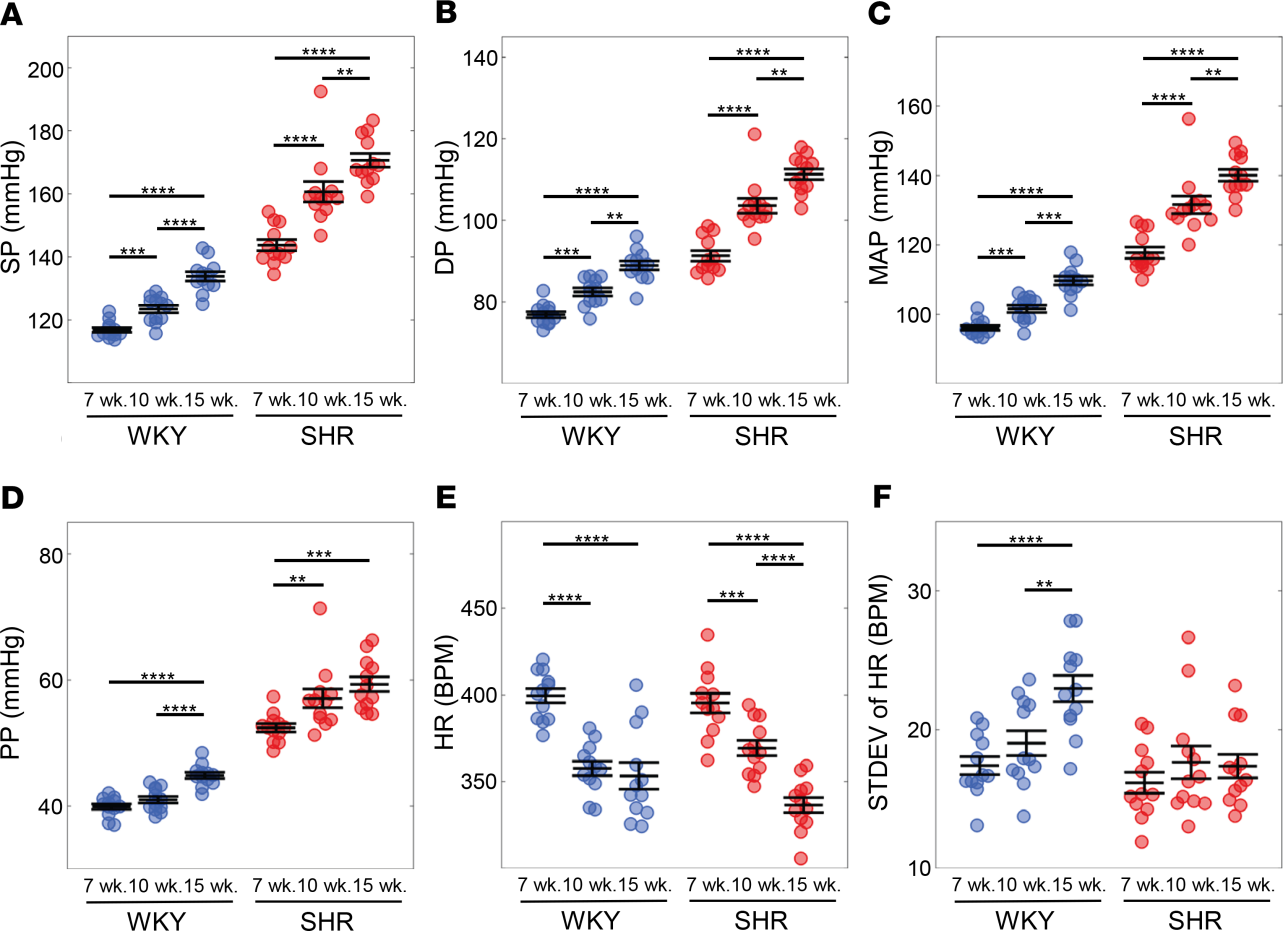
Arterial pressure increases with age in the SHR and WKY rats. Arterial pressure was measured continuously over the 12-hour dark cycle at 7, 10, and 15 weeks of age in SHR and WKY rats. In both strains, (**A**) systolic pressure (SP), (**B**) diastolic pressure (DP), (**C**) mean arterial pressure (MAP), and (**D**) pulse pressure (PP) steadily increased with age. The overall MAP in the SHR was higher than in the WKY. (**E**) Heart rate (HR) decreased with age in both strains. (**F**) Standard deviation (STDEV) of the HR increased with age in the WKY and not in the SHR. Data are shown as mean ± SEM with *n* = 12 animals per group. Statistical comparisons were made using a 2-way ANOVA and Holm-Šídák multiple-comparisons test: **P < 0.01, ***P < 0.001, ****P < 0.0001. WKY, Wistar-Kyoto rat; SHR, spontaneously hypertensive rat.

Pressure measurements revealed systolic pressure (SP), diastolic pressure (DP), MAP, and pulse pressure (PP) in the SHR were all significantly (*P* < 0.0001) elevated compared with the WKY rats for both sexes and at all ages studied (Figure 1, A–D). All pressure measurements, SP, DP, MAP, and PP, increased with age in the both SHR and WKY. HR decreased with age in both SHR and WKY rats (Figure 1E). The standard deviation of HR, which was used as an estimate of HR variability, remained unchanged with age in the SHRs and increased with age in the WKY rats (Figure 1F). Data illustrated in Figure 1 are summarized in Table 1. As reported in Table 1 and Supplemental Figure 1 (supplemental material available online with this article; https://doi.org/10.1172/jci.insight.139789DS1), elasticity of the carotid artery was significantly higher in the SHR compared with the age-matched WKY rats, whereas no differences were found in elasticity of the aorta.

Data from 6 male and 6 female animals are combined in Figure 1. The only differences in pressure measurements associated with sex were in DP and MAP in the SHR group at 7 weeks, with average pressures in females slightly higher than in males (*P* < 0.05, Supplemental Figure 2, A–D). Females also tended to have higher HR than age-matched males for both strains, but the differences were statistically significant only for the SHRs at 10 weeks and the WKY rats at 15 weeks (*P* < 0.05, Supplemental Figure 2E). No sex-linked difference was found in the standard deviation of HR (Supplemental Figure 2F) or in arterial mechanics (Supplemental Figure 1, C and D). Because sex-associated differences were minor and arterial pressures in adult animals were not different between sexes, data from both sexes were pooled in the subsequent results and analysis.

### Evaluation of baroreflex intermittency.

**Figure 2:**
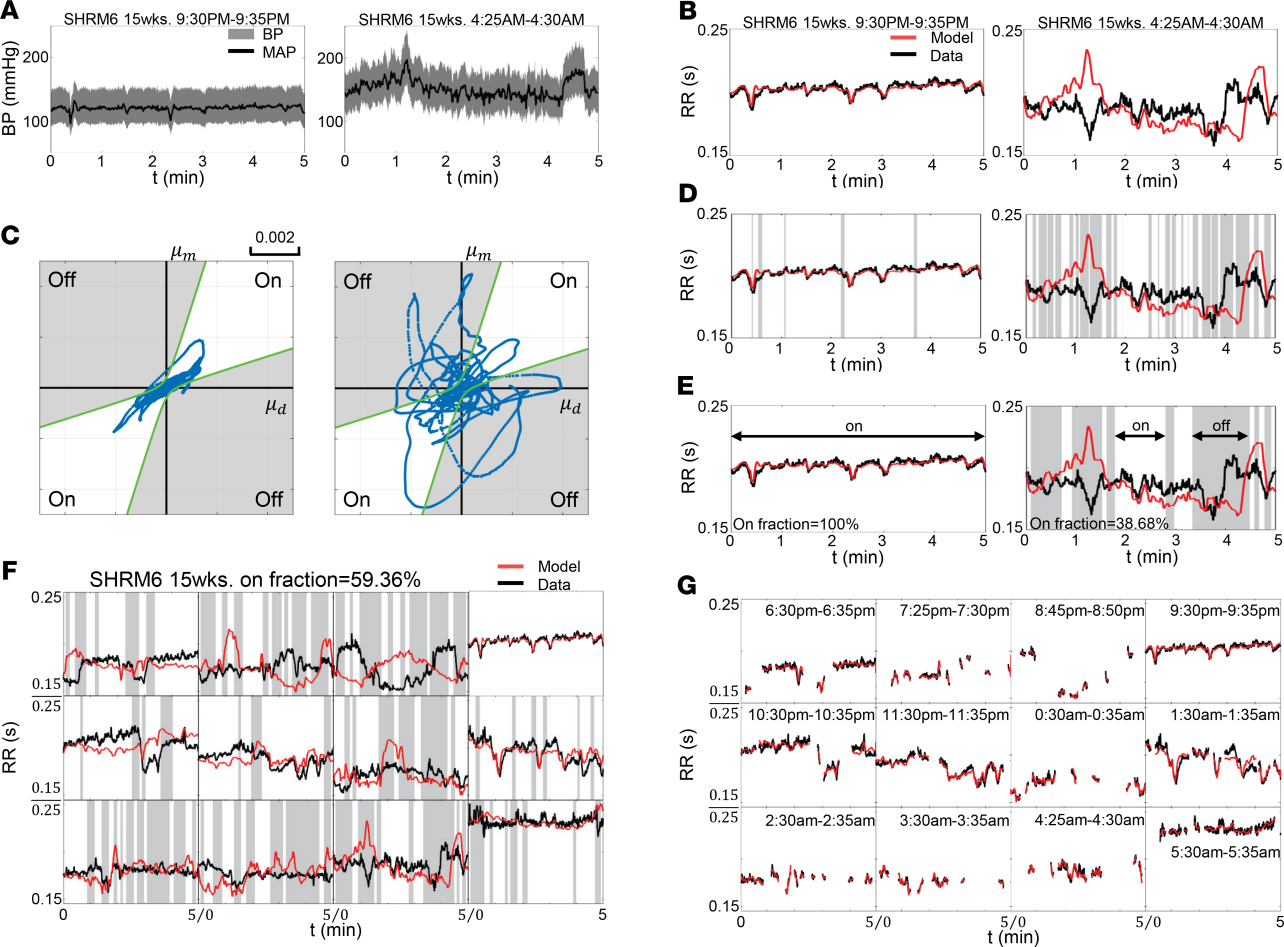
Identification of baroreflex on and off states. (**A**) Direct conscious arterial BP measurement taken at 9:30 p.m. to 9:35 p.m. and 4:25 a.m. to 4:30 a.m. from a 15-week-old male SHR (SHRM6). (**B**) Corresponding RR interval (RR = 1/HR) measured by the telemetry (RR_data_: black lines) versus model prediction (RR_model_: red lines). (**C**) The change of RR interval per unit time reflected in data (μ_d_) is plotted versus change in RR interval per unit time predicted by the model (μ_m_). Data points located between the boundary curves (green) are classified as corresponding to the on state (white area), while the others are classified as corresponding to an off state (gray area). (**D**) Observed and model-predicted RR interval are plotted for the two 5-minute time windows with on state (white) and off state (gray). (**E**) Observed and model-predicted RR interval are plotted for the two 5-minute time windows with times in off state colored gray after filtering to remove noise. (**F**) Observed and model-predicted RR interval are plotted for the twelve 5-minute time windows recorded over a 12-hour dark cycle with times in off state colored gray for SHRM6 at 15 weeks old. (**G**) Model predictions (red) are compared with data (black) during on-state times for the twelve 5-minute windows.

The workflow for identifying intermittent on and off states of the baroreflex arc from continuous arterial pressure data is illustrated in Figure 2. The methodology is described in detail in the Methods section. Two examples of continuous 5-minute time-course BP data obtained from a 15-week old male SHR are shown in Figure 2A, while corresponding data on RR interval (RR = 1/HR) over the same time windows are shown as solid black curves in Figure 2B. The RR data are compared with fits (solid red curves) to a simple mathematical model of the baroreflex arc in Figure 2B. When the baroreflex was controlling HR, as in the left panels of Figure 2, A and B, transient reductions in MAP resulted in transient reductions in RR interval (reflecting an increase in HR); transient peaks in MAP are followed by transient peaks in RR interval. The dynamic coupling between MAP and RR interval is reflected in a close match between the model and the data in the left panel of Figure 2B. For the time window illustrated in the right panels of Figure 2, A and B, the MAP and RR interval are not coupled in a way that would be predicted by physiological operation of the baroreflex. For example, transient pressure increases are followed by transient increases in HR. The lack of physiological coupling of the baroreflex is reflected in the lack of a match between model simulations and data in the right panel of Figure 2B. To identify periods when baroreflex control of HR is and is not functioning, in Figure 2C we plotted the rate of change of RR interval predicted by the model (μ_m_) versus the rate of change observed in the data (μ_d_). Points that fall inside the boundary curves (white region) in these plots are identified as periods when the baroreflex is functioning. Points that lie outside the boundary curves (gray regions) are identified as periods when there was no coupling between fluctuations in BP and HR. The corresponding off states for the time series shown in Figure 2, A and B, are colored gray in Figure 2D. Noise in the signal was filtered out, resulting in the final determination of on and off states for these time windows, as illustrated in Figure 2E.

Figure 2F illustrates the identified on and off times for twelve 5-minute windows recorded over a 12-hour dark cycle for an individual rat. It is shown that for some 5-minute periods the baroreflex appears to function in the on state for the full 5-minute window. For others the baroreflex is assessed to be in the off state for most of the window.

### On fraction decreases with hypertension and aging in SHR and WKY rats.

**Figure 3:**
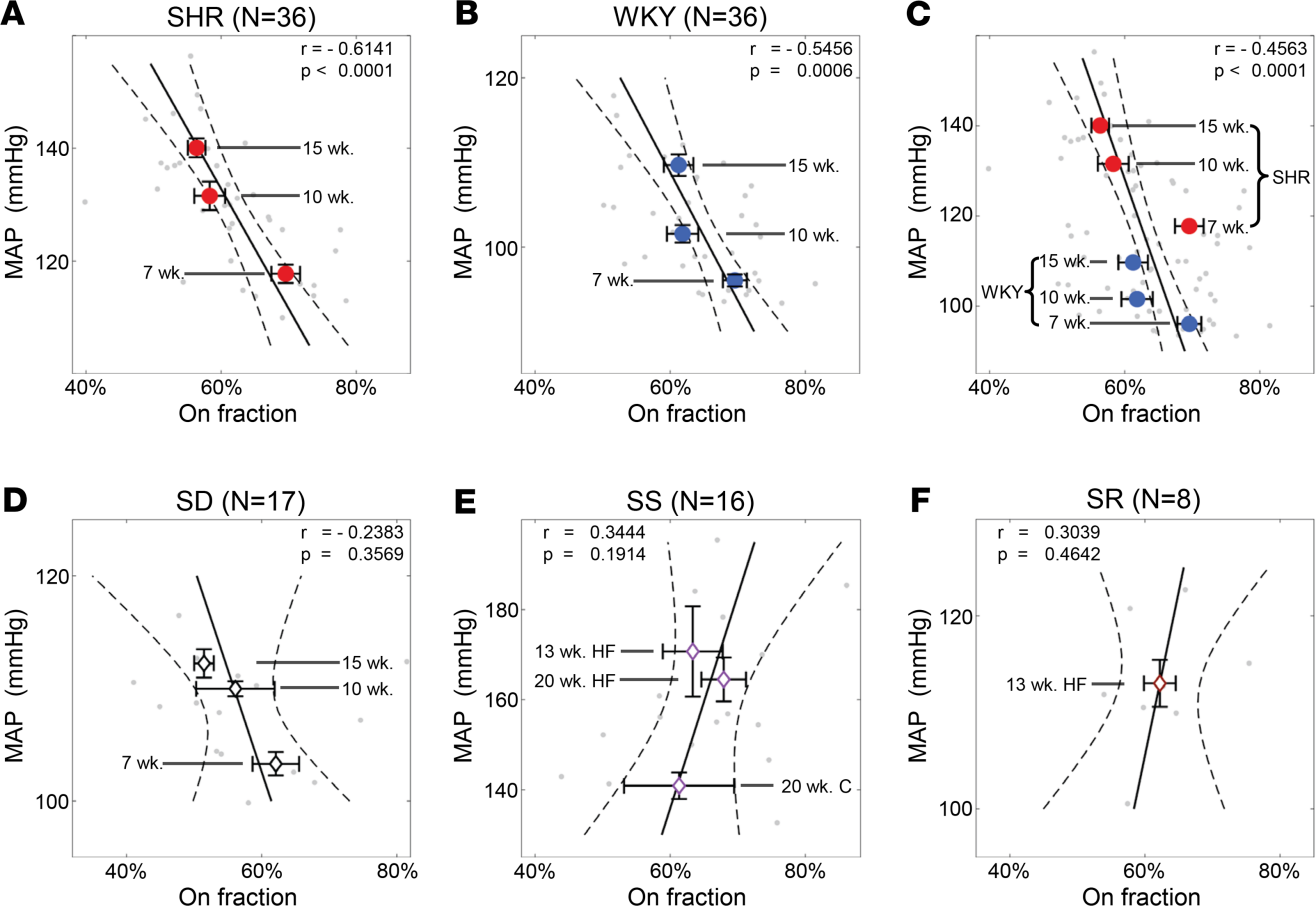
On fraction decreases with aging and development of hypertension in the SHR/WKY rats. Gray dots in each panel represent measured MAP and estimate on fraction for an individual animal at an individual age. (**A** and **B**) There is a significant negative correlation between the MAP and on fraction in the SHR and WKY strains. On fraction decreases with age in both the SHR and the WKY strains. (**C**) When data from SHR and WKY animals are pooled, there is a significant negative correlation between MAP and on fraction, indicating that the on fraction is strongly predictive of MAP in the SHR/WKY strains. (**D**–**F**) There is no significant correlation between the MAP and on fraction in the SD, SS, and SR strains or between age and on fraction in the SD and SS strains. Data are shown as both the individual value and mean ± SEM. Correlation is determined by Pearson’s correlation coefficient (*r*). Observed correlation is reported as significant if *P* < 0.05.

Figure 3, Table 1, and Table 2 summarize data on the relationships between baroreflex function, hypertension, and age. The estimated baroreflex sensitivity (*α*) was not found to be different between any of the strains or ages studied (Table 1). No correlations were found between baroreflex sensitivity and MAP, arterial mechanics, or age. However, the on fraction was found to decrease with MAP and age (Figure 3, A and B) in both the SHR and WKY rats. Furthermore, the on fraction was tightly correlated with MAP in the data set combining data from SHR and WKY rats (Figure 3C). The average on fraction was lower in the SHR than in the WKY at 15 weeks (Supplemental Figure 3). In sum the on fraction is strongly predictive of MAP and the degree of hypertension in SHR and WKY rats.

Given that the SHR and WKY rats share a similar origin and genetic background (19), we investigated whether the predictive relationship between on fraction and MAP is a universal phenomenon or unique to the SHR/WKY genetic background. Results from analogous analysis performed on the SS, SR, and SD rats are illustrated in Figure 3, D–F. Results from conscious direct BP measurements in SD rats at 8, 11, and 15 weeks of age showed no significant relationship between MAP and on fraction (Figure 3D). Data from hypertensive SS rats (with and without high-fat feeding) and from normotensive control diet SS rats also showed no significant relationship between MAP and on fraction (Figure 3E). Finally, data from normotensive SR rats on a high-fat diet also showed no such relationship (Figure 3F). Thus, among the animal models studied here, only the SHR and WKY strains showed a predictive relationship between baroreflex on fraction and MAP.

### MAP in the SHR and WKY rats is higher and tends to increase during off state compared with on state.

**Figure 4:**
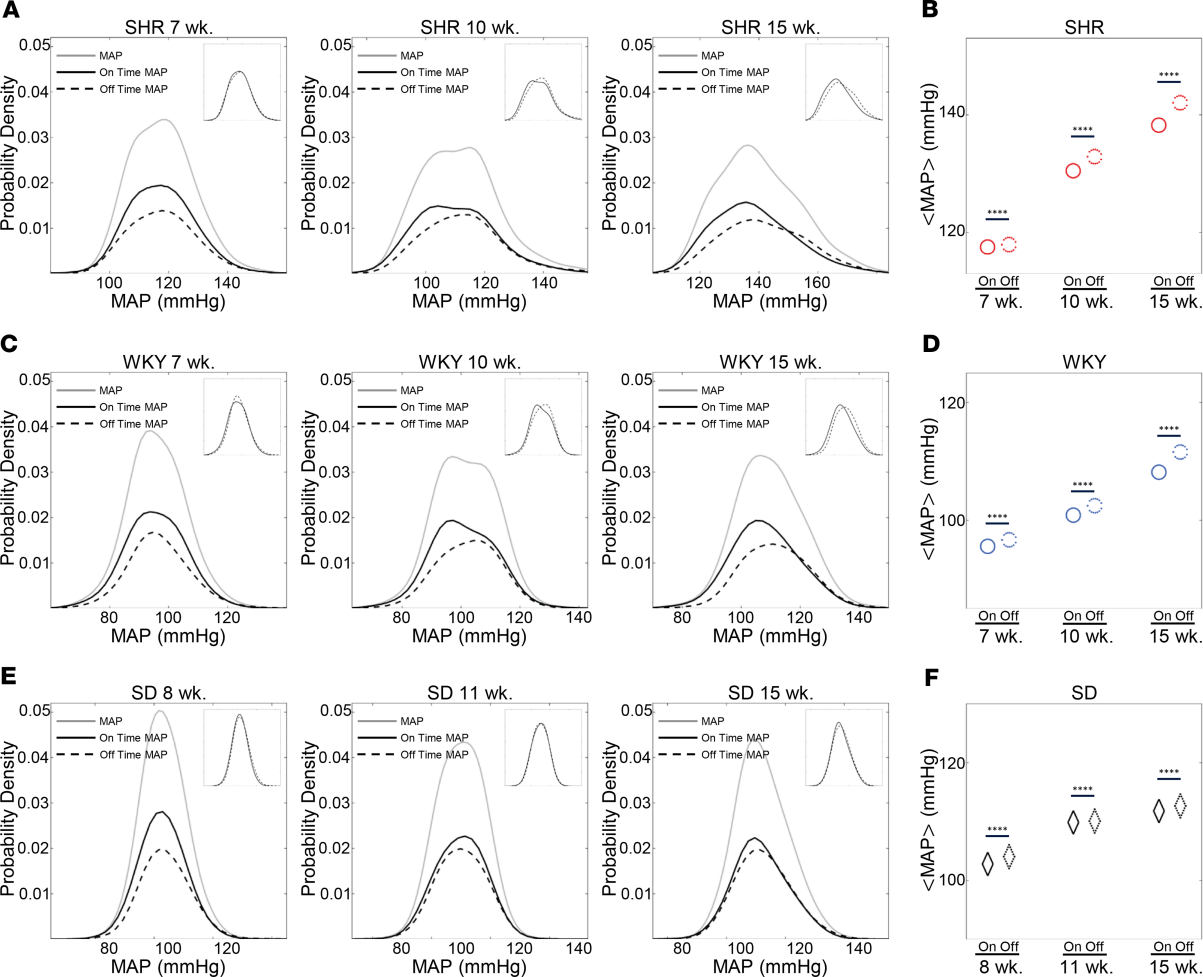
The average level and lability of the MAP are lower during the on state compared with the off state in the SHR/WKY strains. (**A**, **C**, and **E**) The gray solid lines represent the probability density distribution of the total MAP recording. The black solid lines represent probability density distributions of MAP during the on state, whereas the black dashed lines represent probability density distributions of MAP during the off states. The inset figures plot the MAP probability density functions normalized to have unit area for both on- and off-state distributions. The average level of MAP increases with age in the SHR and WKY, as shown in panels **A** and **C**. Furthermore, both the mean level and variability in MAP are lower during on states compared with during off states in the SHR and WKY (**A** and **C**) but not in the SD (**E**). The difference in average pressure increases with the progression of hypertension and aging. (**B**, **D**, and **F**) The mean values of the probability distributions of MAP for 8-, 10-, and 15-week SHR, WKY, and SD rats during baroreflex-on and -off states are plotted. Data are shown as mean. Unpaired 2-tailed Student’s *t* test, ****P < 0.0001.

To probe how on fraction affects BP regulation in the SHR/WKY model, we constructed probability density distributions of MAP observed during baroreflex on and off states. Figure 4 plots probability densities of MAP measured at the per-beat level during on and off states in SHR, WKY, and SD strains at the 3 ages studied. In SHR and WKY animals the pressure distribution widened with increasing age, indicating larger fluctuations in pressure compared with younger animals. The widest distribution (most variable MAP) was observed for the 15-week SHR. Figure 4, B, D, and F, plot the average MAP values obtained during on and off times at different ages and in the different strains. (MAP was calculated here per beat and thus fluctuates, as indicated in the figures. The average MAP is denoted as <MAP>.) In all rat strains and at all ages, <MAP> was higher (*P* < 0.0001) during baroreflex-off times than it was during on times. However, the observed difference in <MAP> between on and off times became greater than 1.6 mmHg only for the SHR and WKY groups at ages 10 and 15 weeks. In the age-matched SD rats, the difference in <MAP> was always less than 0.9 mmHg. Similarly, small differences were observed for the data from SS and SR rats (data not shown). Although 1.6 mmHg is a small difference, a tendency for relatively small increases during frequent intermittent off times could contribute to a shift to larger differences over chronic time scales.

**Figure 5:**
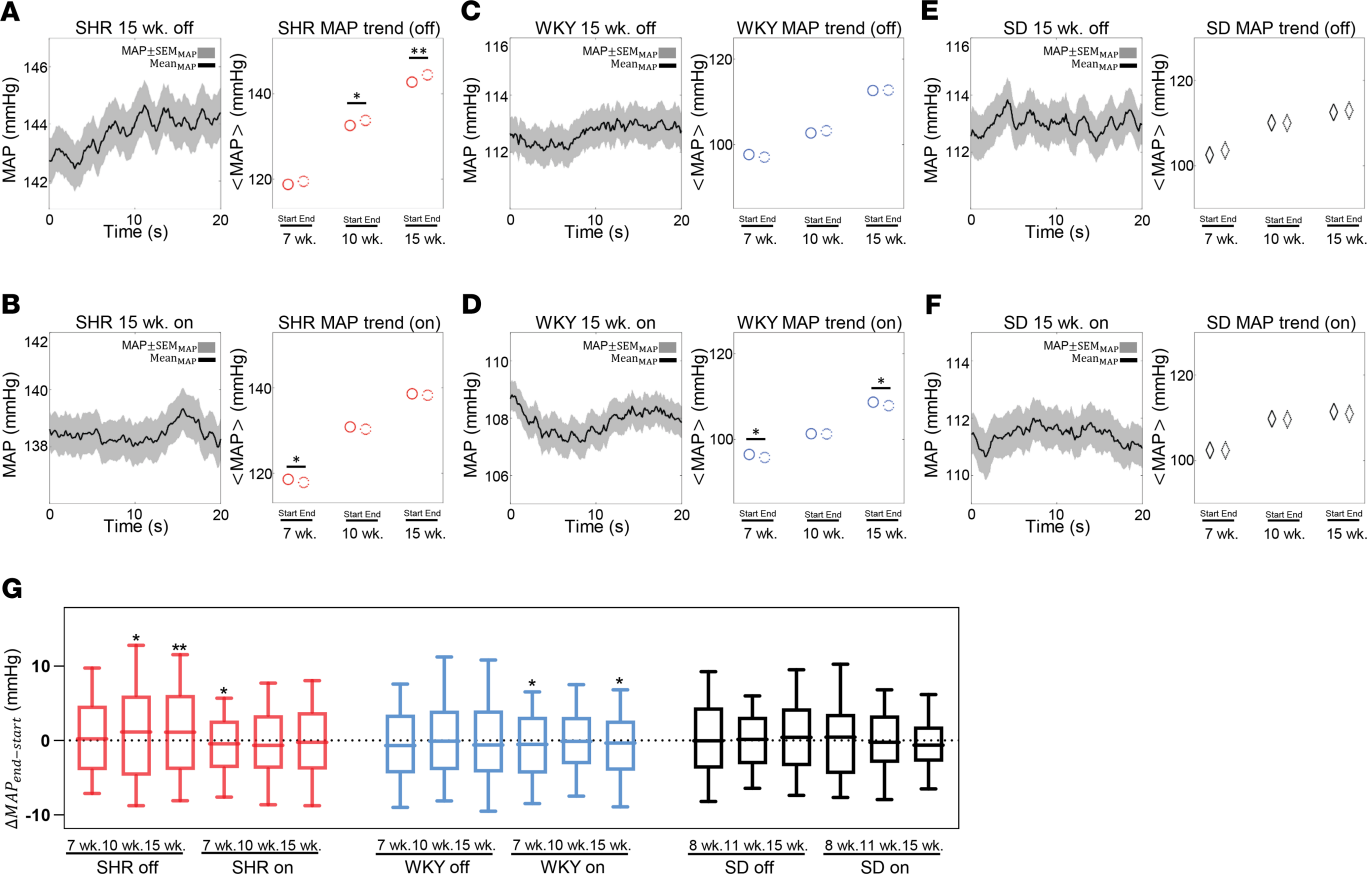
Temporal trends in MAP during on and off states. Trends in MAP recorded during all on and off states longer than 20 seconds are illustrated for 15-week-old SHR animals during baroreflex-off states (**A**) and -on states (**B**), for 15-week WKY animals during baroreflex-off states (**C**) and -on states (**D**), and for 15-week SD animals during baroreflex-off periods (**E**) and -on periods (**F**). In these time series, time 0 represents the onset of the switch to an on or off state. The solid black curves represent the mean trends for each case and the gray area represents ±1 SEM. The averages of MAP at the beginning and after 20 seconds in the off state are plotted for 7-, 10-, and 15-week SHR (**A**), WKY (**C**), and SD (**E**) animals. The averages of MAP at the beginning and after 20 seconds in the on state are plotted for 7-, 10-, and 15-week SHR (**B**), WKY (**D**), and SD (**F**) animals. The MAP tends to decrease during the first 20 seconds in on states and increase during the first 20 seconds in off states in SHR and WKY animals. These trends are not apparent in SD animals. Changes in pressure observed over the first 20 seconds of off and on states are summarized for SHR, WKY, and SD animals at 7, 10, and 15 weeks of age (**G**). The rate of pressure increase during off states increases and the rate of pressure decrease during on states decreases with age in the SHR. In panel **A** through panel **F**, data are shown as mean ± SEM. In panel **G**, data are shown in box-and-whisker plots (10%–90%). Paired 2-tailed Student’s *t* test: *P < 0.05, **P < 0.01.

Figure 5 plots time courses of MAP during the first 20 seconds of baroreflex-on and -off times in SHR, WKY, and SD rats at 15 weeks of age. Although there was a high degree of variability in the individual time courses of MAP, the average trend (solid black curves in the time-series figures) showed an increase with time during off times in the 15-week SHRs (Figure 5A). Similarly, there were decreases in the average trend during on times in the 15-week SHR and WKY animals (Figure 5, B and D). Overall, MAP tended to increase during the off state and decrease during the on state in both the SHR and WKY strains, with statistically significant increases in MAP during the first 20 seconds of off states that were observed for SHRs at 10 (*P* < 0.05) and 15 (*P* < 0.01) weeks. Statistically significant decreases in MAP during the first 20 seconds of on times were observed for SHRs at 7 (*P* < 0.05) weeks and WKY rats at 7 (*P* < 0.05) and 15 (*P* < 0.05) weeks. No significant trends were observed for SD rats at any age. The changes in MAP observed during the initial 20 seconds of baroreflex-on and -off times are summarized in Figure 5G.

Summarizing the results plotted in Figure 4 and Figure 5: (a) both the MAP and the MAP variability were higher during baroreflex-off state than during the on state in only SHR and WKY rats; (b) during on and off times of at least 20 seconds, the MAP tended to increase in the first 20 seconds during the off state and decrease during the on state only in the SHR/WKY rat model; (c) in the SHR strain, the amount that the MAP decreased during the first 20 seconds of on states became smaller with age and the onset of the hypertensive phenotype, while the amount that the MAP tended to increase during the initial 20 seconds of off states increases with age.

### Chronic BP increases more in the WKY following baroreflex ablation than in the SHR.

The preceding data suggest that differences in the intermittent function of the baroreflex between the SHR and WKY rat contribute to determining differences in MAP between these strains. Specifically, the hypothesis is that the more frequent engagement of baroreflex in the WKY rat compared with the SHR contributes to the lower pressure in the WKY compared with the SHR. This hypothesis was tested by performing sinoaortic denervation to ablate baroreflex afferent signaling in WKY and SHR animals at 6 weeks of age. If the greater engagement of baroreflex in the WKY is partly responsible for lower MAP in the WKY compared with the SHR, then ablation of the baroreflex would cause a greater increase in MAP in WKY than in SHR.

**Figure 6:**
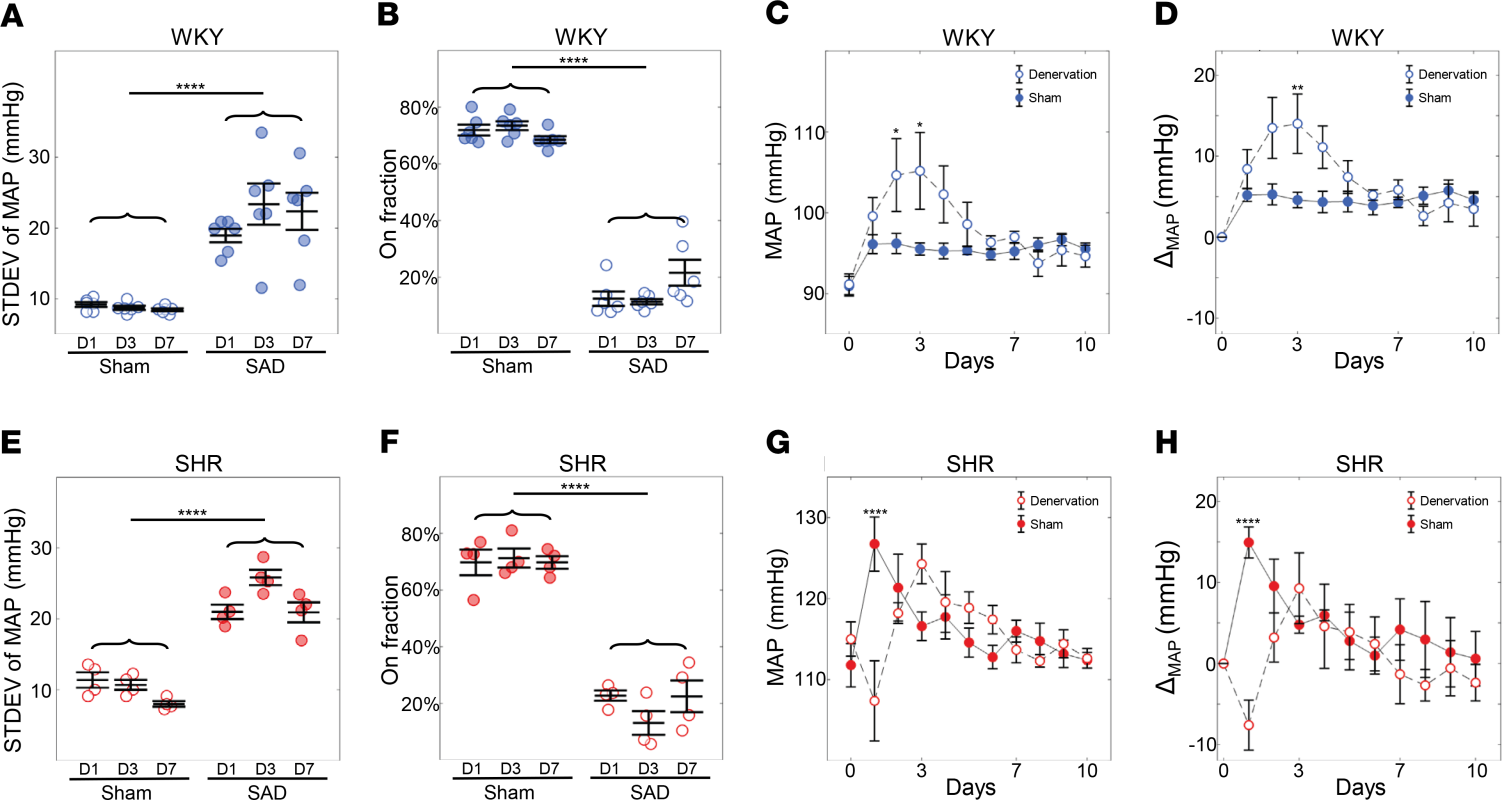
Increase in MAP in the WKY following SAD surgery. MAP is reported as the average over the whole dark cycle at each day. MAP for day 0 is reported as the 5-day average of the MAP before SAD. The SAD was performed on day 1 and pressure data recording continued. The standard deviation of MAP was significantly higher in the SAD group compared with the sham group both in the WKY (**A**) and SHR (**E**). The on fraction in the SAD group is significantly lower compared with the sham group in both the WKY (**B**) and SHR (**F**). Combining these indicated the SAD procedure was successful. MAP measured in the WKY rats following sham (*n* = 6) and SAD (*n* = 6) surgeries. (**C** and **D**) MAP remains elevated in the SAD group compared with sham for the first 7 days following surgery with a peak average MAP of 105.2 mmHg for the SAD group at day 3 compared with 95.53 mmHg for the sham group. Panel **D** plots the change in MAP relative to the pressure at day 0. (**G** and **H**) MAP measured in SHR following sham (*n* = 4) and SAD (*n* = 4) surgeries. For the SHR, there was no increase in MAP in the denervation group compared with sham group. Panel **H** plots the change in MAP relative to the pressure at day 0. Data are shown as mean ± SEM with *n* = 6 for the WKY and *n* = 4 for the SHR. Statistical comparisons were made using 2-way ANOVA, Holm-Šídák multiple-comparisons test: *P < 0.05, **P < 0.01, ****P < 0.0001.

Measurements of MAP from dark cycle conscious monitoring for 10 days after the implantation of the telemetry device (5 weeks of age) and sinoaortic denervation (SAD) surgery (6 weeks) are summarized in Figure 6. As expected, the variability in MAP (the standard deviation of MAP) increased in both the WKY-SAD and SHR-SAD groups following SAD surgery (Figure 6, A and E), verifying the success of the baroreflex ablation. Furthermore, the estimated baroreflex on fraction for the SAD group was substantially and significantly lower than in sham rats, which is consistent with an ablated baroreflex (Figure 6, B and F). Daytime BP waveforms from 15-week-old SHR-sham and WKY-sham rats were also analyzed to evaluate baroreflex intermittency. Although the on fraction in the light cycle was significantly higher than dark cycle (Supplemental Figure 4A), the on fraction estimated for the light cycle was highly correlated to that for dark (Supplemental Figure 4B).

Consistent with observations from other animal models and rat strains (21–23), MAP in the WKY-SAD was elevated compared with WKY-sham over the first several days following denervation (Figure 6, C and D). However, MAP in the SHR-SAD group did not show any increase compared with the SHR-sham group (Figure 6, G and H). At day 3 following denervation, the MAP was 9.4 mmHg (*P* < 0.01) higher in the WKY-SAD compared with the WKY-sham group. At this age, the difference in MAP between the SHR and WKY groups was approximately 21.1 mmHg. Thus, at 3 days following SAD surgery, the pressure in the WKY-SAD group had increased by roughly half of the difference observed between the WKY and SHR groups. In other words, half of the pressure elevation that occurred in the SHR compared with the WKY was attained in the WKY following baroreflex denervation. At no point did the MAP in the SHR-SAD group show any significant elevation compared with the SHR-sham group. After approximately 5 days, the pressure in the WKY-SAD group returned to the same mean value as the WKY-sham group, presumably because of compensation by other BP control mechanisms.

In summary, chronic changes in BP in the WKY-SAD compared with the SHR-SAD are consistent with the hypothesis that more frequent engagement of baroreflex in WKY rats compared with SHR contributed to the lower pressure in the WKY compared with the SHR. These changes lasted for several days, suggesting that differences in baroreflex function between the WKY and SHR contribute to differences in MAP in these strains.

## Discussion

The current studies reveal that (a) during normal dark cycle recording the fraction of time the baroreflex control of HR spent in the on state (the “on fraction”) ranged from 40% to 85% for all rat strains observed; (b) the on fraction decreased with age only in the SHR and WKY strains; (c) the MAP was tightly correlated with on fraction in only the SHR and WKY strains; (d) mean pressures during off state were higher than during on state in the SHR and WKY strains for both sexes and at all ages studied; and (e) increases in pressure in the first few days following SAD were higher in WKY compared with the SHR. As discussed below, these observations are consistent with the potentially novel hypothesis that the differences in *intermittent functioning* of the baroreflex play a causal role in the development of hypertension in the SHR strain and the established but still controversial hypothesis that differences in baroreflex function between the WKY and the SHR are partially responsible for the differences in arterial pressure between these strains.

Arterial BP regulation is achieved via the interaction of multiple organs and organ systems (14), including the autonomic nervous system. Overactivity of the sympathetic nervous system has been observed in the patients with hypertension for decades (24–26). The classical interpretation of the role of baroreflex control of autonomic activity and BP is that it does not contribute to long-term regulation over time scales substantially greater than that of the heartbeat (1, 8, 13). It is widely accepted that the stiffening of large arteries that is associated with hypertension blunts the sensitivity of the arterial (carotid and aortic) baroreceptors and, thus, decreases baroreflex sensitivity (16, 18, 27). Impaired functioning of the baroreflex has been observed in both animal models (28, 29) and hypertensive patients (24, 30). While the potential for baroreflex dysfunction to play a causal role in the etiology of hypertension is controversial (8, 31), the success of carotid baroreceptor activation therapy for treating resistant hypertension (12, 31–37) would be hard to explain if the baroreflex did not play a role in long-term arterial pressure regulation. In addition, knockout of mechanically activated channels (PIEZOs) that play crucial roles in baroreceptor afferent strain sensing results in an approximately 7.0 mmHg increase in MAP compared with WT mice (38).

The present study reveals a potentially new mechanism by which the baroreflex may control arterial pressure in the SHR model of primary hypertension. Specifically, observations of time series of arterial BP fluctuations in conscious ambulatory animals reveal the existence of apparent intermittent engagement (on state) and disengagement (off state) of baroreflex-mediated HR control in the rat. This robust and reproducible algorithm identifying on and off states and estimating on and off fractions reveals that periods of effective disengagement (off states) can last for several minutes, or thousands of heartbeats, in rats. Furthermore, analysis of the intermittent on and off states reveals that the fraction of time spent in the off state is positively correlated with MAP in the SHR/WKY rat and not correlated with MAP in other strains of hypertensive and normotensive rats. Moreover, the MAP is higher and tends to increase during off states compared with on states in SHR/WKY rats but not in other strains. These observations, and the effects of sinoaortic denervation on MAP in SHR and WKY rats (discussed later), are consistent with the hypothesis that dysfunction of baroreflex contributes in a unique way to the etiology of hypertension in the SHR.

Numerous techniques exist for assessing baroreflex function based on measuring responses to externally imposed changes in pressure. For example, the Valsalva maneuver elicits a drop in transmural pressures in the aortic arch and carotid arteries, generating a reflex-mediated increase in HR, which may be analyzed by a variety of mathematical techniques (39). Studies in animals and humans have also used administration of vasodilators and vasoconstrictors to bring about arterial pressure and corresponding HR changes to assess the baroreflex response (40, 41). The intermittent functioning of the reflex response that we observed during normal conscious baseline conditions would not be seen in studies relying on methods where large transient changes in arterial pressure are imposed.

A critical question is, What is the cause of the baroreflex intermittency that we document here? Although our time-series analysis reveals intermittent periods where the baroreflex appears not to be controlling HR, we do not interpret the identified off times as representing periods when either afferent or efferent components of the baroreflex are completely nonfunctional. Rather, we speculate that during identified off times, central nervous system mechanisms are transiently overriding the baroreflex control of HR as they do during bouts of exercise, mental stress, or changes in respiration. Observations of pressure fluctuations during baroreflex-on and -off states in the SD, SS, and SR strains reveal that, while the phenomenon of apparent intermittency of the baroreflex control of HR is not unique to the SHR and WKY strains, the statistical associations between baroreflex function and MAP are unique to the SHR and WKY. Thus, the intermittency phenomenon does not likely arise as a secondary sequelae of hypertension.

While the intermittency phenomenon was seen in all strains of normo- and hypertensive rats we studied, the behavior of the SHR and WKY rats was distinguished from the other strains by the predictive relationship between on/off fraction and MAP and the apparent trends in MAP that were observed during the transient on and off times. This could be related to the relative role of the autonomic nervous system in BP regulation in the various models we studied. For example, although baroreflex impairment and neurogenic mechanisms contribute to the BP differences between Dahl SS rats on different salt intakes (42), autonomic nervous system abnormalities appear to play no role in high-fat diet–induced hypertension in Dahl SS rats (43–45). This is in contrast to the SHR, where autonomic factors clearly are important for the maintenance of hypertension.

We observed that MAP in the WKY-SAD became equivalent to that of the WKY-sham group after several days postsurgery, consistent with previous observations in rats and other small animals (29, 46). This result appears to argue against a critical function of the baroreflex in chronic pressure regulation in this strain. But the phenomenon may result from a compensatory response from other BP control mechanisms. It does not appear to result from pressure-diuresis (47), but rather it has been observed that the initially elevated renal sympathetic nerve activity following SAD in Wistar rats eventually returns to normal levels (48). Thus, changes in MAP following SAD appear to follow changes in sympathetic nerve activity. We speculate that this restoration of sympathetic activity and MAP could be a central adaptation to withdrawal of afferent baroreceptor input lasting several days or more.

The SHR and WKY strains share a similar origin because they are outbred from the Wistar rat (49). Although the genetic backgrounds of these 2 strains are not identical (50, 51), the WKY is categorized as the best genetically normotensive control rat for comparison to the SHR (19, 51). Congenic studies have revealed that because the alleles specifically enriched in SHR were common variants already present in WKY from which it was derived (51), genetically the SHR arguably represents the selection of the combination of the worst prehypertensive traits of the WKY.

To summarize the unique features of the SHR/WKY strains: (a) they share a common genetic background; (b) the relationships between intermittent HR control and MAP are apparent for only the SHR/WKY strains; and (c) ablation of baroreflex function in the less hypertensive WKY strain leads to increases in MAP. We speculate the WKY and the SHR strains have an enhanced reliance on the baroreflex for long-term pressure control, perhaps because of some shared defect among the other parallel physiological pathways regulating arterial BP. Moreover, MAP increases with an age-associated deterioration of baroreflex function in these animals. This deterioration is delayed in the WKY compared with the SHR. Based on this interpretation, we propose that the search for genetic underpinnings of hypertension in this model should focus both on genetic differences between the SHR and WKY (52–54) and on genetic variations shared by the SHR and WKY but not with other strains.

Further research is needed to investigate if and how these findings translate to hypertension in the human population. Previous investigations into the intermittent engagement of the baroreflex have focused on very short-term transients identified using a baroreflex effectiveness index (BEI), which is a measurement of the percentage of beat-to-beat changes in SP that are followed by a change in HR (55). It has been shown that abnormalities in BEI are associated with cardiovascular disease (56, 57). Yet because the sequence method to estimate BEI is influenced by breath state and is difficult to reproduce (58), and because it does not identify periods of disengagement lasting longer than 3 or 4 heartbeats, its application would not identify the relatively long periods of disengagement identified in this study.

The algorithm developed here has the potentially unique and novel ability to identify relatively long periods (lasting on the order of several minutes) during which the baroreflex system is and is not operating in a manner in which fluctuations in arterial pressure are coupled to corresponding fluctuations in HR. Because the on fraction metric distinguishes between different etiologies of disease in different rat models of hypertension, the metric may therefore be useful in distinguishing between different etiologies of hypertensive disease in humans. Given the trends of increasing pressure during off states and decreasing pressure during on states are observed only in the SHR/WKY strains, the etiology of hypertension in the SHR may be representative of patients with labile hypertension (59). Furthermore, we speculate that baroreflex on fraction may have the potential to predict the responsiveness to carotid baroreceptor stimulation therapy in patients with resistant hypertension and/or be useful in characterizing autonomic disorders, such as diabetic autonomic neuropathy, familial dysautonomia, and postural orthostatic tachycardia syndrome.

## Methods

### Rats strains, care, and use

#### Rat strains.

Data for time-series analysis of arterial pressure were obtained from experiments at University of Michigan (U-Mich) and at Michigan State University (MSU). Telemetry-based recordings of arterial pressure in rats at U-Mich used the following study group: 5-week-old male and SHRs (*n* = 12) and 5-week-old male and female WKY rats (*n* = 12) purchased from Charles River Laboratory, as well as 6-week-old male SD rats (*n* = 6) bred in the Unit for Laboratory Animal Medicine of U-Mich. Telemetry-based recordings of arterial pressure in rats at MSU used the following study groups: 13-week-old male Dahl SS rats (*n* = 8) and 13-week-old male Dahl SR rats (*n* = 8) purchased from Envigo and fed a high-fat diet, as well as 20-week-old female Dahl SS rats purchased from Charles River Laboratory and fed either a high-fat diet (*n* = 4) or control diet (*n* = 4). Baroreceptor denervation studies were performed at U-Mich using 5-week-old male and female SHRs (*n* = 8) and 5-week-old male and female WKY rats (*n* = 8) purchased from Charles River Laboratory.

#### Diet protocol.

SS and SR rats at MSU were fed a high-fat diet or control diet. High-fat diet (D12492 fat) was from Research Diets and was composed of 60% fat, 20% protein, and 20% carbohydrate. Control diet (D12450J) was composed of 10% fat, 20% protein, and 70% carbohydrate.

#### Conscious direct measurement of arterial pressure.

A radio-telemetry system (Data Sciences International) was used to collect real-time arterial pressure waveforms data at U-Mich. To implant the telemeter (PA-C10), anesthesia was induced by 5% isoflurane in oxygen and maintained by an adjusted (1.5%–2%) concentration. With the aid of a dissecting stereomicroscope, a 6–8 cm abdominal midline skin incision was made with scissors. The intestines were retracted with a saline-moistened sterile sponge to expose the infrarenal aorta. The catheter of the PA-C10 was inserted into the infrarenal abdominal aorta, and the body of the implant was secured to the right vertebral muscle just caudal to the right kidney with nonabsorbable sutures (4-0 braided polyester). No mortalities occurred during surgery. Most animals were fully ambulatory within 30 minutes and returned to regular drinking, eating, and bowel function within 1 hour. After a 7-day recovery, the arterial pressure was recorded continuously (500 Hz) for the whole dark cycle (6 p.m. to 6 a.m.). SHR and WKY were recorded at 7, 10, and 15 weeks of age. Data from the SD animals were recorded at 8, 11, and 15 weeks of age. A radio-telemetry system (Data Sciences International) was used to collect real-time arterial pressure waveforms data at MSU. Anesthesia was performed using the described procedure. To implant the telemeter (HD-S10), the skin on the inner left thigh was opened to expose the femoral vessels. Blunt dissection was used to form a subcutaneous pocket overlying the caudoventral abdomen, and the transmitter was placed into this pocket. The catheter of an HD-S10 was introduced into the vessel and advanced beyond the iliac bifurcation until the pressure-sensing tip was situated in the abdominal aorta. No mortalities occurred during surgery. After a 14- to 21-day recovery, the arterial pressure was recorded continuously (500 Hz) for the whole dark cycle (6 p.m. to 6 a.m.) at 13 (SS and SR males) and 20 weeks of age (SS females).

#### Measurement of central arterial elasticity.

The elasticities of the aorta and left carotid were measured by using a combination of EKV-mode ultrasonography system with an MS250 transducer for arterial wall thickness (Vevo 2100, Fujifilm VisualSonics, Inc.) and carotid BP measured from the radio-telemetry system. Rats were anesthetized as previously described (60). Two-dimensional arterial ultrasound images of aorta and carotid were obtained near the aorta arch and left carotid sinus area in the supine position. The images obtained were analyzed by using Vevo Vasc Analysis software (version 2.2.3; Fujifilm VisualSonics, Inc.) to estimate the systolic thickness (T_sys_) and diastolic thickness (T_dia_). The elasticity (E) was calculated according to this formula: E = (P_sys_ – P_dia_) × (T_dia_/T_sys_ – T_dia_).

#### SAD and sham surgery.

The SAD procedure was modified from the previously reported method (61). Rats were anesthetized by 5% isoflurane in oxygen and maintained by an adjusted (1.5%–2%) concentration. An anterior midline neck incision was performed followed by careful retraction of the sternohyoid and sternocleidomastoid muscles. The carotid bifurcation was exposed, and the occipital artery was retracted. For aortic baroreceptor denervation, the aortic depressor and superior laryngeal nerves were observed and sectioned. For carotid baroreceptor denervation at the carotid sinus, the surrounding nerves were stripped and chemically denervated using 10% phenol in 100% ethanol. Carotid denervation was executed bilaterally. Sham-operated rats underwent the same surgical procedures to expose the carotid sinus, but all nerves were left intact.

### Computational analysis of pressure and HR time-series data

Time-series analysis of the telemetry data is based on the assumption that when the HR is controlled through the baroreflex, changes in arterial pressure should effect changes in HR; i.e., transient increases/decreases in MAP and/or PP should be followed by proportional decreases/increases in HR. The simple linear filter-based approach of Kosinski et al. (39) was modified to distinguish time windows when the baroreflex is functioning (“on”) or is not functioning (“off”). In other words, “off” represents times when the expected relationship between BP and HR does not exist. Though the specific formulation of this model is not crucially important, we chose the Kosinski model for its simplicity. An alternative mathematical model is likely to give equivalent results for the present applications as long as the alternative model captures the central phenomenon that a change in BP effects an inversely proportional change in HR.

#### Time-series data collection and processing.

Conscious BP was recorded continuously during the active dark cycle (6 p.m. to 6 a.m.). Because of occasional glitches in the telemetry system, the full 12-hour raw BP data sets were occasionally corrupted by missing data. To avoid the influence of missing data, we selected one 5-minute time course of uncorrupted data in each 1-hour block. Hence, we collected 12 individual 5-minute BP time courses representative of each hour of the 12-hour raw data for analysis. These 5-minute periods were selected as close as possible to the middle of each hour. SP and DP were taken as the local maxima and minima of the PP signal, and MAP was approximated at each cardiac cycle *t_j_* for *j* = 1, …, *N* (*N* the total number of cardiac cycles in each 5-minute period) as

 (Equation 1).
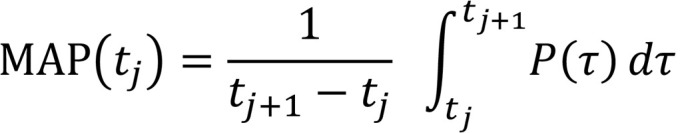


Figure 2A shows 2 examples of continuous 5-minute time-course pressure data obtained from an SHR at 15 weeks.

#### Run time environment.

All algorithms were developed and implemented in MATLAB 2019b. All codes and raw data are available via doi 10.5281/zenodo.3778991 or at https://github.com/ebenjaminrandall/IntermittentBaroreflex

#### Analysis of continuous BP and HR data.

Data on RR interval — RR(*t*), HR =1/RR — were compared with predictions from a linear filter similar to the model used by Kosinski et al. (39) analyzing the autonomic response to the Valsalva maneuver in human subjects. Using the MAP data as an input, the model assumes that the RR interval determined by the autonomic baroreflex is captured by the first-order linear filter:

 (Equation 2),
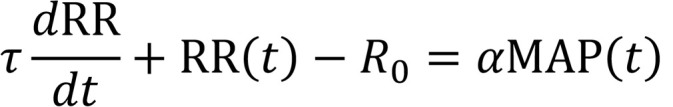


where α (s/mmHg), *τ* (s), and R_0_ (s) are parameters. Values for *τ* and α are given in Table 2, and R_0_ was calculated as R_0_ = <RR> – α<MAP>,

 (Equation 3),
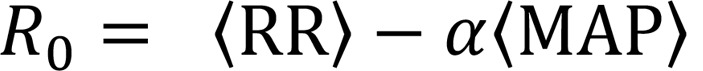


where <·> indicates the mean value over a given 5-minute time course. In Equation 2, *α* represents the baroreflex sensitivity measured in units of change in RR interval per unit change in BP. The time constant *τ* determines how quickly changes in BP will generate changes in HR (the inverse 1/*τ* is the response rate). Assuming a piecewise constant right-hand side of Equation 2 over an individual beat of duration Δ*τ*, the solution to Equation 2 is

 (Equation 4).
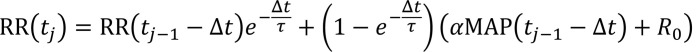


This approach yields substantial variability in the ability of the model to match the data, as illustrated in Figure 2B, which shows an example of the model fitting the data well (left panel) and poorly (right panel). An assessment of the poor model fits suggests an intermittent engagement and disengagement of the baroreflex-mediated control of HR. During periods when the RR interval data change in the direction predicted by the model, we classify these as when the baroreflex arc is functioning as expected or in an on state. We identify periods when the model does not coincide with the data as representing baroreflex-off states.

#### Estimation of parameters.

To determine the time constant τ and gain α for each subject at a given age, we minimized the cost functional (*J*) computing the relative difference in the variability of the HR between the model output and the data. We quantified this variability by computing the slope of the line of regression within a fixed window T centered at cardiac cycle *t_j_* during a 5-minute time course I = 1, …, 12 for both the model output (μ_m,I,j_) and the data (μ_d,I,j_). Thus, μ_m,I,j_ and μ_d,I,j_ are unitless values reflecting the local rates of change in RR interval per unit time. We simultaneously fit all twelve 5-minute data sets obtained from a given animal at a given age. For this study, we chose T = 10 seconds, though equivalent results are obtained when T is set to half or twice this value. The standard deviation of the slopes of the model output σ_m_ = std(μ_m_) and data σ_d_ = std(μ_d_) were computed for

 (Equation 5).
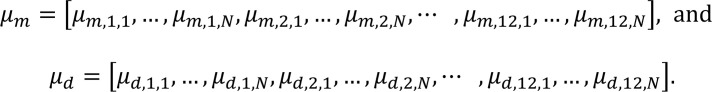


Hence, we minimize the cost functional

 (Equation 6)
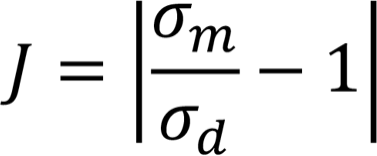


to obtain optimal parameter values for τ and α for each subject at each age.

#### Identification of on and off periods.

To compare the model-predicted change in RR interval to the observed data and determine on and off states from the BP time-course data, we located regions in the plot of the slopes μ_m_ versus μ_d_ (Figure 2C) for which the linear response model did not predict the observed response, that is, when the points lie away from the *y* = *x* line. The boundary for the on and off domains is demarcated by the hyperbola

 (Equation 7),
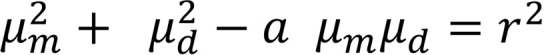


which has symmetry about *y* = *x*. The parameter *r* is the magnitude of the *x*- and *y*-intercepts, and α is a parameter determining the curvature of the hyperbola. To exclude relatively small fluctuations in μ from being identified as representing off states, the parameter *r* was set to 1/2σ_d_ of all the rats (*r* = 0.0004 for normal rats and *r* = 0.00015 for denervated rats). The unitless parameter α was set to a value of 3.5. The computed trends were not sensitive to the choice of α. In plotting μ_m_ versus μ_d_, we prescribed the on state to points that fall inside the hyperbola (white region) bounded by the hyperbola. The points that lie outside the hyperbola (gray regions) were considered off. Figure 2D displays the on and off states for the BP time course given in Figure 2, A and B.

#### Noise filtering.

We applied a smoothing procedure to filter out noise in the sequences of on and off designations. A cardiac cycle *t_j_* was initially assigned a value of 1 if it is on and 0 if it is off. The resulting time series was smoothed by iteratively computing the 10-second moving window average of the data until the result converged. After smoothing, time points that are less than 0.5 are off and at least 0.5 are on. Figure 2E illustrates the final off and on states after smoothing the results from Figure 2D.

#### On fraction.

We define the index on fraction as on fraction = total time in on state/total time, which describes the fraction of time when an individual animal at a given age is in the on state.

#### Statistics.

Representative probability distributions and correlation statistics were computed using the MATLAB ksdensity and polyfit functions. Statistical analyses were performed using GraphPad Prism 8.1.1, such as using an unpaired 2-tailed Student’s *t* test to analyze normally distributed data and a 2-way ANOVA corrected by the Geisser-Greenhouse method and Pearson’s correlation coefficient to make multiple comparisons. Levels of statistical significance are indicated as *P < 0.05, **P < 0.01, ***P < 0.001, and ****P < 0.0001.

#### Study approval.

Experiments were performed under permission and guidelines of the Institutional Animal Care and Use Committees of U-Mich and MSU.

## Author contributions

FG, SW, BEC, DEM, and DAB conceived the study. FG, EBR, BEC, and DAB developed the computational methodology. FG and EBR were responsible for the baroreflex mathematical model and algorithm for baroreflex intermittency. FG collected data and did formal analysis. GDF, DEM, and DAB provided data resources. FG and SW carried out animal surgeries. KCB performed animal ultrasound measurements. FG wrote the original article draft, which EBR, SW, GDF, DEM, and DAB reviewed and edited. BEC, GDF, DEM, and DAB supervised.

## Supplementary Material

Supplemental data

## Figures and Tables

**Table 1:**
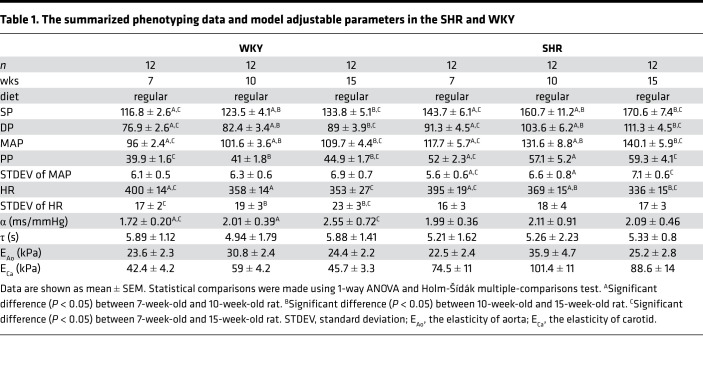
The summarized phenotyping data and model adjustable parameters in the SHR and WKY

**Table 2:**
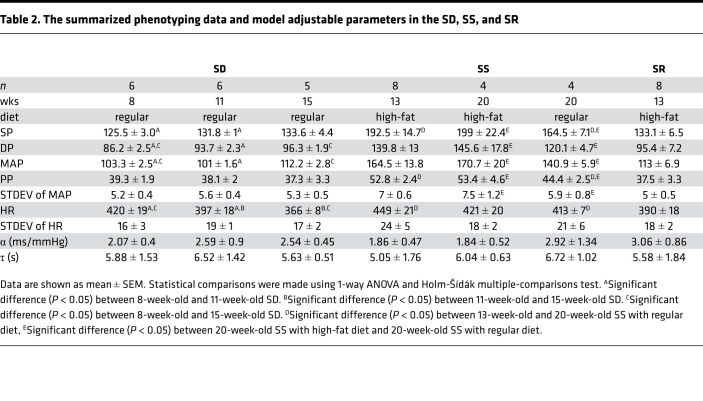
The summarized phenotyping data and model adjustable parameters in the SD, SS, and SR

## Version 1 10/02/2020

Electronic publication
