# Supplementary material for: Potential role of intermittent functioning of baroreflexes in the etiology of hypertension in spontaneously hypertensive rats
Source: JCI Insight. 2020 Oct 2;5(19):e139789. doi: 10.1172/jci.insight.139789 (PMC7566704; doi:10.1172/jci.insight.139789)
Supplement: Supplemental data [file jciinsight-5-139789-s134.pdf]

Supplemental Material for: Potential role of intermittent functioning of baroreflex in the etiology of hypertension in spontaneously hypertensive rats

Feng Gu[1, 2], E. Benjamin Randall[1], Steven Whitesall[1], Kimber Converso-Baran[1], Brian E. Carlson[1], Gregory D. Fink[3], Daniel E. Michele[1], Daniel A. Beard[1, *]

[1]Molecular and Integrative Physiology, University of Michigan, Ann Arbor, Michigan, USA
[2]Vascular surgery, Second Xiangya Hospital, Central South University, Changsha, Hunan, China
[3]Pharmacology and Toxicology, Michigan State University, East Lansing, Michigan, USA

*Corresponding Author
Tel: 734 763 8040
Address: 2800 Plymouth Road, University of Michigan, Ann Arbor, MI
email: beardda@umich.edu

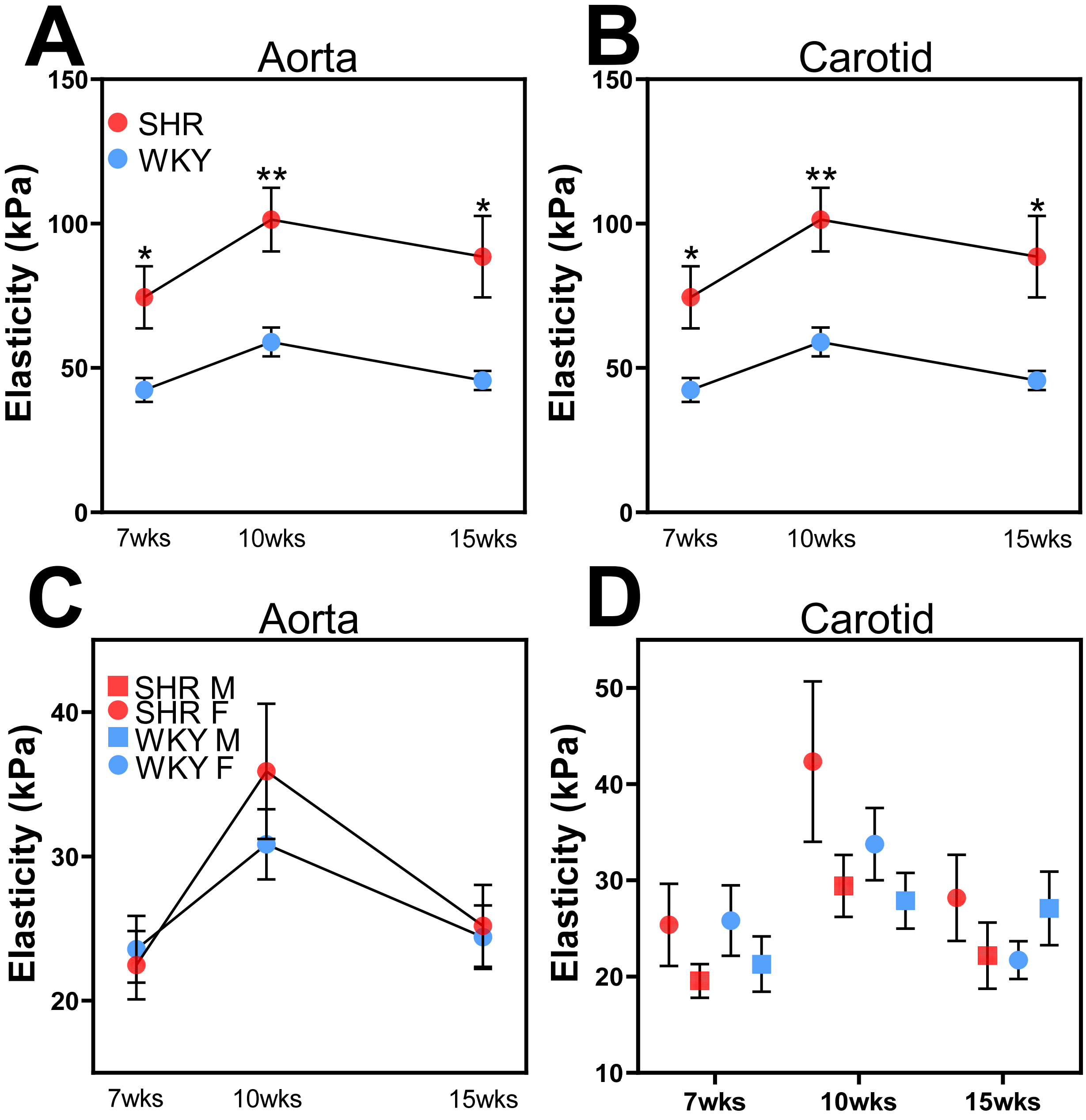

Supplementary Figure 1: (A, B) Arterial mechanics do not change with aging and hypertension in the SHR (red) and WKY (blue) rats. The carotid arterial wall of the SHR was stiffer than that of the WKY but no difference was observed for the aortic wall. (C, D) No sexual difference of arterial mechanics was found. The square represents male and the circle female. Data measured by vessel ultrasound are shown as mean ± SEM with n=12 or 6 animals per group. Sidak's multiple comparisons test, *$p < 0.05$. WKY, Wistar-Kyoto rat; SHR, spontaneously hypertensive rat.

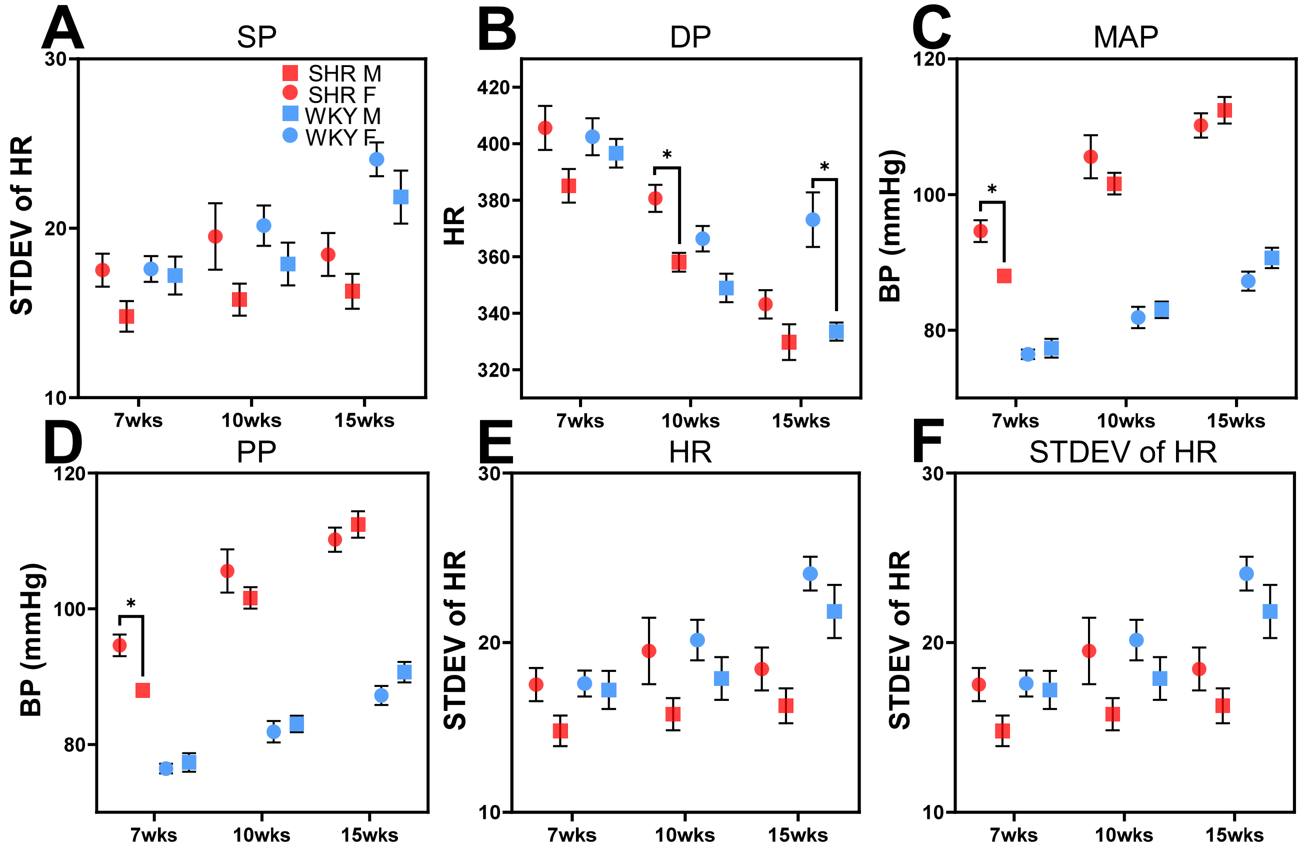

Supplementary Figure 2: Sex-linked differences in phenotyping data from the SHR (red) and WKY (blue) rats. Square symbols represent males and circles represent females. Data which were measured by telemetry are shown as mean ± SEM with n=6 animals per group. Statistical tests use Sidak's multiple comparisons test with * indicating $p < 0.05$. WKY, Wistar-Kyoto rat; SHR, spontaneously hypertensive rat; M, male; F, female; BP, blood pressure; HR, heart rate.

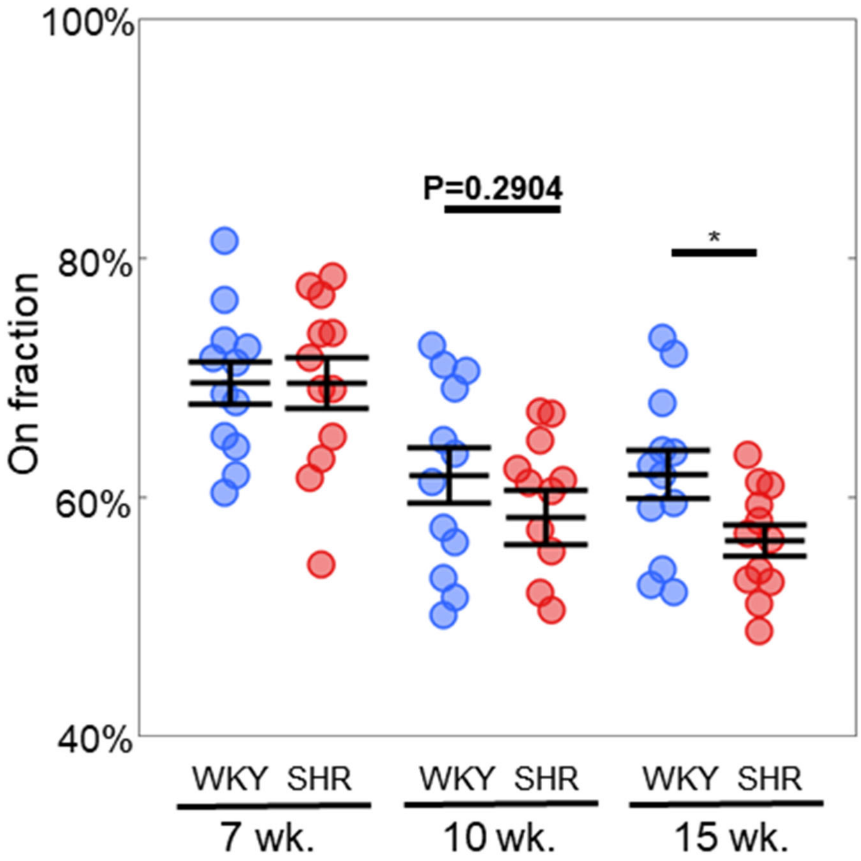

Supplementary Figure 3: On fraction in the SHR strain is lower than the WKY strain. There is no difference in on fraction between the SHR and WKY at 7 weeks old. The WKY tends to have higher on fraction than the SHR in 10 weeks old but not significant (p =0.2904) and becomes significant when the animal becomes 15 weeks old. Data are shown as mean ± SEM, with n=12 animals per group. Statistical comparisons are made using unpaired T-test: $*p < 0.05$. WKY, Wistar-Kyoto rat; SHR, spontaneously hypertensive rat.

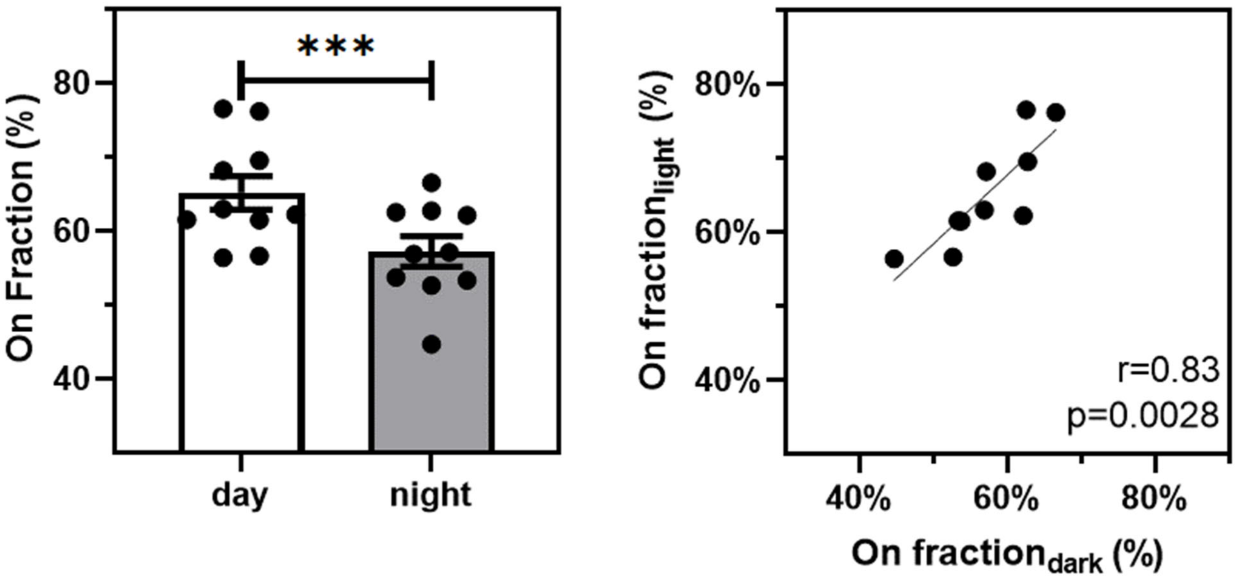

Supplementary Figure 4: Baroreflex intermittency analysis of BP waveform from 12h-dark cycle and 12h-light cycle from 15 weeks old SHR/WKY-sham rats. (A) On fraction of light cycle is higher compared to dark cycle. (B) The on fraction between dark cycle and light cycle are highly correlated. Data are shown as mean ± SEM, with n=10 animals per group. Statistical comparisons are made using paired T-test: ***$p < 0.001$, Observed correlation is reported as significant if $p < 0.05$. WKY, Wistar-Kyoto rat; SHR, spontaneously hypertensive rat.
